# Morphological, structural, chemical, vibrational, thermal, pasting, and functional changes in pea starch during germination process

**DOI:** 10.12688/f1000research.136568.2

**Published:** 2025-09-11

**Authors:** Juan Carlos Lucas-Aguirre, Víctor Dumar Quintero-Castaño, Luisa Fernanda Castañeda-Cano, Mario Enrique Rodríguez-García

**Affiliations:** 1 Facultad de Ciencias Agroindustriales, University of Quindio, Armenia, Quindio, 630001, Colombia; 2 Department of Nanotechnology, Center for Applied Physics and Advanced Technology. National Autonomous University of Mexico. Campus Juriquilla, Querétaro. C.P. 76230, Mexico, National Autonomous University of Mexico National, Mexico City, Mexico City, Mexico

**Keywords:** pea, starch, germination, starch nanocrystals, pasting.

## Abstract

**Background:**

Changes in the structural and physicochemical properties of pea starch could be significantly affected by germination treatment, which can provide a theoretical basis for promoting the use of this starch in the food industry.

**Methods:**

This work aims to evaluate the effect of germination time on the structural, thermal, rheological and functional properties of pea starches to determine their potential in the production of fermented beverages. The physicochemical changes during the germination process of peas and native and germinated starch granules were evaluated.

**Results:**

For germination critical time was 0.985 days, with 95% of germinated grains. The starch grains did not undergo any morphological change during the germination process as seen in the scanning electron microscopy images, indicating the absence of the α and ß - amylases responsible for the starch splitting. The X-ray patterns revealed that the crystalline structures of pea starch with and without germination were unchanged and contained by hexagonal and orthorhombic glucopyranose crystals. The viscosity profiles of the starches do not show significant changes; the most representative change is the increase in the gelatinization onset temperature of the paste from germinated starches compared to native starch. The functional properties of starches showed generally low values, with statistically significant differences between water absorption index, water solubility index, and starch swelling power and germination time.

**Conclusions:**

In general terms, it can be concluded that pea starch does not undergo significant changes in its physicochemical and functional integrity with respect to the grain germination process.

## Introduction

To improve the nutritional value of legumes, preparation techniques have been developed to significantly increase the bioavailability of their nutrients. These techniques include germination, a complex metabolic process that activates and releases enzymes, responsible for changes in the chemical composition of the grain.

Peas ( *Pisum sativum L*.) are legumes recognized for their high nutritional potential, primarily due to their high protein content. They have been proposed as an alternative to soy in countries where peas are not a traditional crop, or in contexts where soy consumption is limited by allergies, intolerances, or other restrictions. Peas contribute to a more sustainable and healthy diet, in addition to reducing dependence on wheat flour and the problems associated with its use.

Currently, there is a growing demand for gluten-free products because gluten can cause autoimmune conditions and immune-mediated reactions in certain individuals. To serve this market, it is ideal to select raw materials that are low-cost, have significant nutritional value, and are rich in proteins, carbohydrates, vitamins, and minerals. For example, the protein content of legume flours varies between 17 and 40 g/100 g on a dry basis, considerably higher than that of cereal flours (3–7 g/100 g, dry basis) health, mainly attributed to the presence of bioactive compounds such as polyphenols ( Urbano *et al.*, 2005; Xu *et al.*, 2019). Furthermore, legume flours contain more dietary fiber than wheat flours and have been used to prepare foods with a lower glycemic index. Thus, partial or total replacement of wheat flour with legume flours may offer additional health benefits and respond to the needs of gluten-sensitive populations. Furthermore, the consumption of legume flours has been linked to improvements in human health, mainly attributed to the presence of bioactive compounds such as polyphenols ( Urbano *et al.*, 2005; Xu *et al.*, 2019).

Germination is responsible for changes in the chemical composition of the grain. Due to which lipids, carbohydrates (starch) and storage proteins within the seed are broken down to obtain the energy and amino acids necessary for the development of the pedicel, roots and at the end of the plant and to be more easily absorbed, which increase the healthy function of the grain ( Urbano *et al.*, 2005; Xu *et al.*, 2019; Aguilar *et al.*, 2020; Gao *et al.*, 2022). Depending on temperature, moisture content, light levels, and grain, the germination stage typically lasts between 4 and 5 days. The grains are rotated occasionally while regulating moisture content and temperature. Enzymes like hemicelluloses, amylases, proteases, oxidases, among others, are engaged. In this stage metabolism is very active ( Oseguera-Toledo *et al.*, 2020). The functional properties of the starch, such as variations in relative viscosity, reducing sugars, apparent amylose, among others, change along with the germination process. As malting progresses so does the capability for water absorption ( Hernández-Becerra *et al.*, 2020).

According to Esquivel-Fajardo *et al.* (2022), starch is a micron or submicron-sized particle made of amylose, amylopectin, water, minerals, salts, and nanocrystals with orthorhombic (A-type starch) or hexagonal crystalline structures (B-type starch). These elements have a significant impact on how the physicochemical properties of starch change during germination. This suggests that it is important to identify the modifications brought about by the germination process in each of these components.

The physicochemical properties of starch after germination can be effectively affected, depending on the type of crop and treatment conditions, but in peas such behavior is largely unknown. Gao *et al.* (2022) reported that the structural and physicochemical properties of pea starch could be significantly affected by germination treatment; these changes could provide a theoretical basis to support the use of this starch in the food industry. However, no information about the starch components affected by germination was reported.

In order to evaluate the potential use of pea flours and starches in food products, this work evaluated the impact of germination time on morphological, structural, thermal, vibrational, sticking and functional properties of pea starch.

## Methods

### Germination process

**Figure 1.**
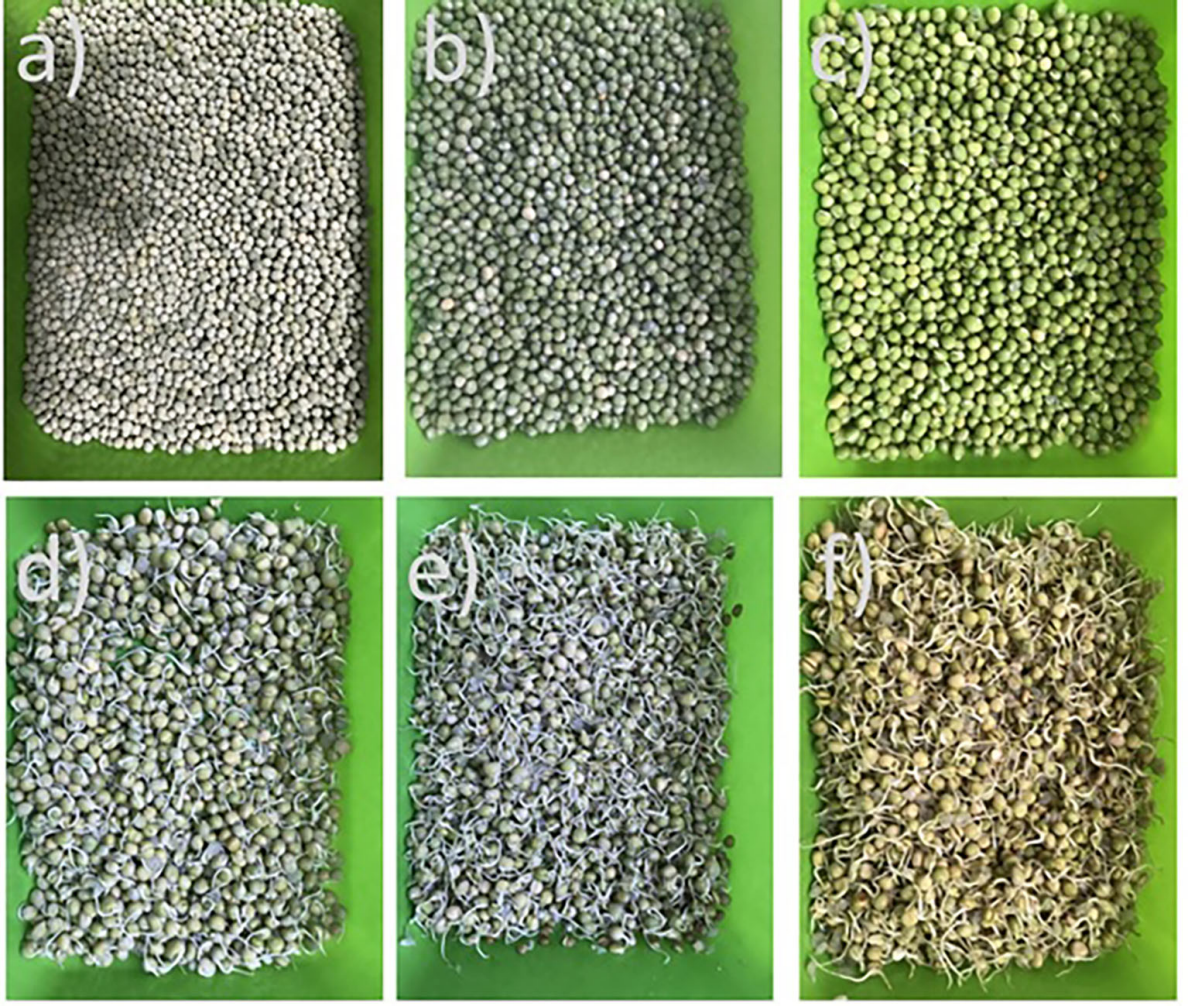
Images of pea a) dry pea, b) soaked pea, c) pea-day 1, d) pea-day 2, e) pea-day 3, f ) pea-day 4 of the germination process.

The San Isidro pea variety, cultivated in Nariño state at the south of Colombia, was used, purchased at the central supply center of the city of Armenia-Quindio. Initially, 1500 g of peas were used, and a manual selection process was carried out to eliminate impurities and broken and/or irregularly shaped kernels. Then, 250 g were weighed for each day of germination for four days and subjected to a 12 hour soaking process in potable water (20°C) using twice the volume of water occupied by the dried peas, during which time rehydration of the peas was ensured. After the extra water was removed, the germination time started and was measured from time zero (0), until it reached 4 days ( Figure 1), the period of time during which the hypocotyl had fully developed.

Every four hours during the four-day germination period, the peas were soaked and flipped over to ensure appropriate humidification and the excess water drained, to prevent the embryo from decaying and dying. Following the conclusion of each germination period, the starch was promptly extracted as explained below.

The germinated flours were produced once the germination process was complete by drying the germinated peas for 48 hours at 40°C in an oven with forced ventilation. To obtain flours, the dry germinated seed were milled using a coffee mill (Krups, Gx550850, Germany) and each one of the flours were then placed in zip-top bags and maintained in a controlled environment of temperature (20°C) and relative humidity (65%). The flour were named as follows: pea flour without germination (PF), the flour for the first day of germination (PF-1), second day (PF-2), third day (PF-3) and fourth day (PF-4). After drying, the flour was ground and sieved through 100 mesh (150 μm).

### Percentage of grain germination

The number of grains that show the existence of a root is determined by obtaining three random samples of 100 grains. Every day, the development of the root was observed, and it was deemed to have germinated when the radicle ruptured the cell wall. When the pedicel was twice the size of the grain, germination had finished. A manual selection of the grains was done to eliminate broken and damaged grains in order to calculate this parameter ( Hernández-Becerra *et al.*, 2020).

### Proximal analysis of germinated flours

Methodologies reported by AOAC (2005) were used for moisture (925.10), lipids (920.39), fiber (978.10), ash (942.05) and protein (920.15), where the moisture content was determined by the oven drying method (105°C, to constant mass); the total fat content was determined by the Soxhlet method; the fiber content by calcination of the dry residue remaining after acid and alkaline digestion of the sample; the ash content by incineration at 550°C; and the protein content was determined by the Kjeldahl method using 6.25 as a factor.

### Starch extraction

The isolated starch both without germination and germinated for each day was obtained following the methodology proposed by Contreras-Jiménez *et al.* (2018) and Londoño-Restrepo *et al.* (2018), where samples were ground with a blender (Krups, KB720, Germany) using distilled water, filtered on a 100 mesh, kept at 4°C for 24 h with distilled water, centrifuged 2270 x g (3000 rpm, Model TD5 Table Top Low Speed Centrifuge, Yingtai Instrument, Changsha, Hunan, China) at 25°C for 15 min, and the clean starch was dried at 45°C for 24 h. After drying, the starch was ground and sieved through 100 mesh (150 μm).

### Amylose content

The amylose content of native and germinated pea starch was determined by the spectrophotometric method (Thermo Scientific ^TM^ GENESYS ^TM^ 150 UV-VISIBLE, Spain) (620 nm) based on the formation of the amylose-iodine complex ( Juliano *et al.*, 1981), for which starch was dispersed in 1 M sodium hydroxide, brought to volume with distilled water (100 ml), the starch was gelatinized and the amylose concentration was determined using an iodine solution (2 g KI + 0.2 g I _2_) against a standard curve with known amylose concentration.

### Morphological changes in the starch

SEM images of the pea isolated starches (PIS) for different germination times were studied using a high vacuum scanning electron microscope (JEOL, JSM-6060LV at high vacuum; Jeol, Tokyo-Japan) with a resolution in HV mode. Each sample was fixed on a specimen holder with carbon tape and sputtered with gold. The analyses conditions used 20 kV electron acceleration voltage at 12–20 Pa pressure ( Quintero-Castaño *et al.*, 2020).

### Particle size distribution (D10, D50, and D90)

Particle sizes were determined as percentiles D10, D50, and D90, using the Mastersizer 3000 (Malvern Instrument Ltd., Worcestershire, UK). The samples were dispersed in 500 mL of distilled water until a darkening value of 10±1% was obtained, considering the size distribution from Mie’s theory and using the refraction index of 1.52 ( Lucas-Aguirre *et al.*, 2019).

### X-ray diffraction analysis

X-ray diffraction patterns of isolated starch along germination process were carried out on a Rigaku-Ultima 4 diffractometer (Rigaku Miniflex, Texas, EE.UU). The operating conditions were 35 kV and 15 mA, with a CuK _α_ radiation wavelength of λ=0.1540 nm, and the measurements were done from 5 to 35° on a 2θ scale. The patterns were carried out using High-Resolution X-ray diffraction with a step size of 0.01° to have a better intensity and resolution of each one of the possible diffracted peaks. The powder samples were packed into an aluminum pan ( Quintero-Castaño *et al.*, 2020). The crystallinity (%) was calculated by normalising the integrated diffracted intensity over the measured 2θ range to the integrated non-coherent intensity. The non-coherent intensity was obtained by subtracting the sharp diffraction peaks from the total diffraction pattern using the software Diffract/AT from Socobin VI.2 ( Contreras-Jiménez *et al.*, 2018).

### Vibrational analysis by FTIR

Isolated pea starches were characterized by FTIR spectrophotometer (Perkin Elmer, Spectrum Two) using ATR (attenuated total reflectance) to identify the main functional groups, and the transmittance was recorded from 600 to 4000 cm ^−1^, with a resolution of 4 cm ^−1^ and 16 scans per sample. Each starch sample was analyzed in triplicate on different samples and the average spectra were reported ( Contreras-Jiménez *et al.*, 2018).

### Thermal properties of starch

The gelatinization temperature and enthalpy of starches obtained from PSI, and isolated starches from each day of the germination process, were analyzed using a differential scanning calorimeter (Mettler Toledo, Greifensee, Switzerland). Calibration was performed using an Indium standard. The samples (2.0±0.1 mg) were prepared by adding deionized water to the samples until they reached 85% moisture (w/w). A similar ratio of starch/water was used for the viscosity measures. The pans were hermetically sealed using a press. Sample scanning was from 30 to 120°C at 7.5°C/min, determining the temperature and enthalpia of starch gelatinization. Each measurement was conducted in duplicate ( Contreras-Jiménez *et al.*, 2018).

### Pasting properties

Apparent viscosity profiles of the PIS, and isolated starches obtained along the germination time, were obtained using a starch cell of an Anton Paar MCR 102 (St Albans, Austria) rheometer following the methodology proposed by Rincón-Londoño *et al.* (2016). Each sample was heated from 50 to 92°C in 5.3 min and then held at a constant temperature of 92°C for another 5.3 min. After that, samples were cooled down to 50°C in 5.3 min and held at that temperature for 1 min. All the tests were carried out at a constant frequency of 3.22 Hz, determining the peak viscosity and the final viscosity after cooling the paste.

### Functional properties of starches

The water absorption index (WAI), the water solubility index (WSI) and the swelling power (SP) of the isolated starches were measured and determined using the methodology reported by Contreras-Jiménez *et al.* (2018), where a 2% W/V starch suspension was heated in a water bath at 90°C for 30 min. The starch samples were centrifuged at 1450 x g (2000 rpm) for 20 min. The supernatant was removed and the sediment was weighed. Aliquots of supernatant were dried in an oven at 100°C to a constant weight, then the respective weights were taken and calculations were performed.

### Statistical analysis

All the analysis were performed following a completely randomized design with three replications. An analysis of variance was performed (ANOVA) with 95% of confidence and Tukey test of mean comparison using Statgraphics Centurion XVIII software.

## Results and discussion

### Percentage of grain germination

**Figure 2.**
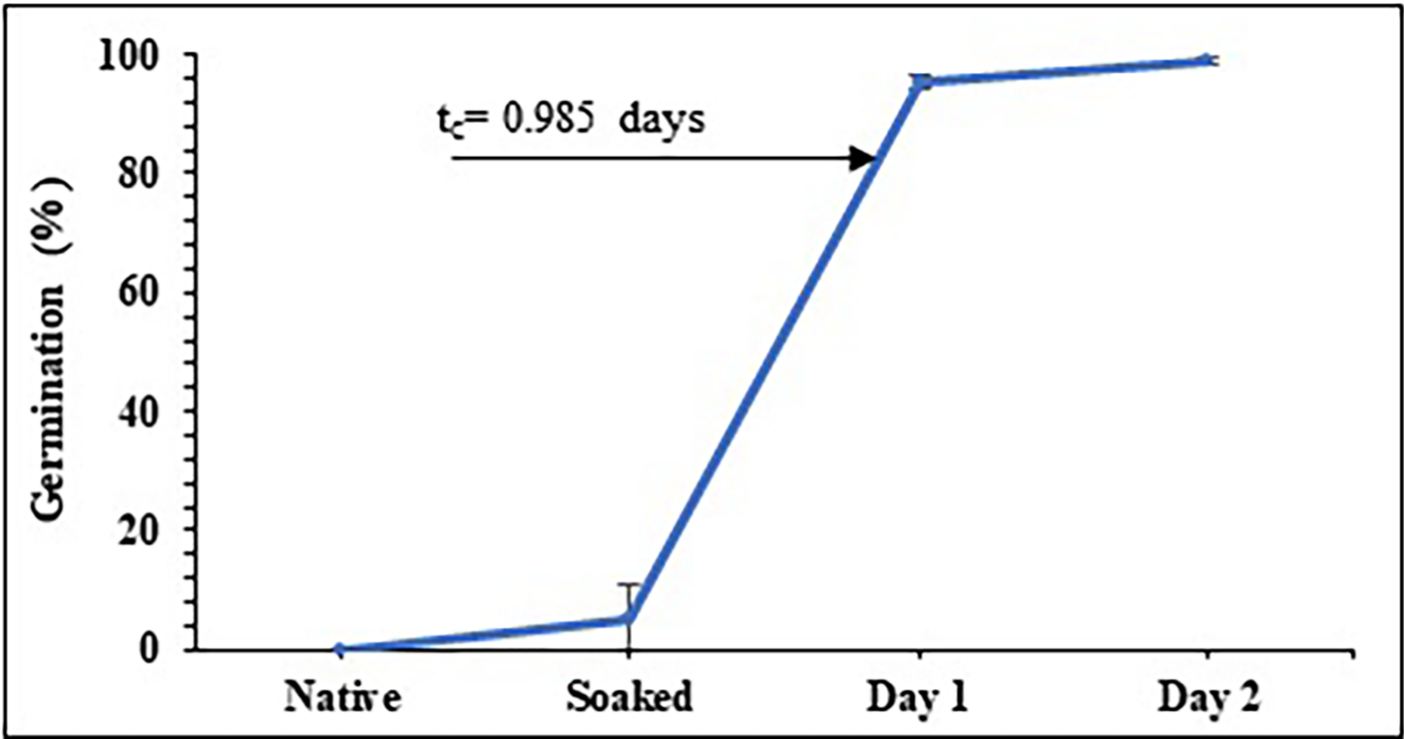
Germination percentage as a function of germination time for pea beans.

Peas were once classified manually with broken, sick, or damaged beans being thrown away. Figure 2 shows the germination percentage as a function of time for the peas, showing that during the soaking time (12 h), the grains reach moisture values from 13.13±0.08% to values close to 40.53±0.52% moisture (wb) and an average of 5 grains germinate ( Figure 1); during the first day of germination, germination percentages of over 95% are reached and over 98% during the second day ( Figure 1).

According to Contreras-Jiménez *et al.* (2018), who studied barley malting; Hernández-Becerra *et al.* (2020), who studied two types of maize; and Oseguera-Toledo *et al.* (2020), who studied sorghum malting, the behavior of germination % exhibits a sigmoidal and/or logistic shape curve. This kind of curve illustrates a second order kinetics in which the system passes through a localized transition between two saturation values. The crucial germination time, or t _c_, is a point at which the shift fundamentally takes place. For pea, t _c_ is around 1 day, but for other products like maize, it is between 1.92 and 1.94 days and for barley, it is between 1.92 and 2.92 days.

As evidenced by the radicle shattering the cell wall on the second day of germination, peas reach their peak value before corn and barley, which germinated on the third and fourth days, respectively. This fact shows that extending of the period for peas boosts their uses when thinking about industrial processes. Other aspects of germination that need to be assessed include the activation of gibberellins, which are phytohormones that cause the interruption of the dormant stage of the seeds and cause them to germinate, and the beginning of latent enzymatic processes with the α and ß-amylases, which use starch and fats as energy sources. These include the grain’s level of hydration. This explains why germination suddenly increased by 60% about t _c_. For two days, gibberellins are created, therefore for the third day decreases ( Gupta *et al.*, 2010; Evans *et al.*, 2009; Bradford *et al.*, 2008).

### Morphological changes during germination

The morphological changes of the pea beans during germination are depicted in Figure 1(a) to (f ). There are no noticeable morphological changes on the surface of the bean after soaking; instead, the bean swelled to nearly double its initial volume, due to hydration of the cotyledons and embryo ( Figure 1(a) and (b)), while from the first day of germination, the hemicellulose enzymes were activated, the cell wall was broken, the radicle emerged ( Figure 1(c)) and the tegument or testa began to separate from the grains. On days 2 and 3, the hypocotyl emerges, which initially remains curved (protected) ( Figure 1(d) and (e)), and continues to grow up to three times the size of the grain. The teste continues to separate more easily and finally on day four, the hypocotyl straightens ( Figure 1f), at which time germination stops and they are dried at 40°C for 48 h.

### Proximal analysis of flours

The proximate composition of the pea flours (PF) and flours obtained from day 1 to day 4 (PF-1 to PF-4) as a function of the germination day is shown in Table 1, presenting statistically significant differences in each of the evaluated variables (p<0.05). In fact, the germination process resulted in a large decrease in total fat content, equal to 74.52%, compared to its value on day 0. As a result of the energy-intensive metabolic activities required for this decrease in fat content, the respiratory rate increased ( Aguilar *et al.*, 2020; Hernández-Becerra *et al.*, 2020; Oseguera-Toledo *et al.*, 2020). Chemical composition is affected by genotype, agronomic conditions, growing conditions such as soil, moisture, temperature, pests, and diseases, and production management ( Oseguera-Toledo *et al.*, 2020).

**Table 1.** Proximal analysis of pea flours with different germination times.

Sample	Moisture (%)	Fat (%)	Fiber (%)	Ash (%)	Protein (%)	Amylose (%)
**PF**	11.21±0.08 ^c^	2.08±0.06 ^de^	6.39±0.52 ^a^	1.68±0.09 ^d^	15.43±0.34 ^a^	18.30±0.02 ^a^
**PF-1**	10.88±0.06 ^b^	1.81±0.09 ^d^	6.80±0.65 ^ab^	1.33±0.12 ^bc^	19.62±0.88 ^b^	18.01±0.04 ^b^
**PF-2**	10.74±0.05 ^b^	1.49±0.04 ^c^	7.20±0.59 ^bc^	1.29±0.08 ^b^	20.07±0.75 ^b^	17.89±0.05 ^c^
**PF-3**	10.02±0.17 ^a^	1.08±0.07 ^b^	7.58±0.73 ^cd^	1.22±0.06 ^b^	21.38±0.08 ^c^	17.23±0.07 ^d^
**PF-4**	11.85±0.45 ^cd^	0.53±0.05 ^a^	8.72±0.85 ^e^	0.84±0.05 ^a^	32.07±0.09 ^d^	18.98±0.12 ^e^

The values correspond to average of proximal content and standard deviation.

Different letters in the same column show significant differences using α = 0.05.

Ash content, which is correlated to the mineral content of the sample, also showed a decrease throughout the germination process, dropping as much as 50.0% by day 4, in comparison to its initial content. Several minerals are needed as coenzymes during germination in order to catalyze the transfer of proteins and carbohydrates to the radicle. These minerals can be lost during the hydration process and/or during germination and can be leached in water during soaking. Consequently, some of these minerals can be lost when the radicles are separated (debarking). The same behavior was seen during the malting of three quinoa varieties by Aguilar *et al.* (2020), and with two maize varieties by Hernández-Becerra *et al.* (2020).

During germination, the protein and fiber contents of the peas increased proportionally by 107.84% and 36.46% respectively, with respect to the initial content. Some proteins can be anabolized from stored carbohydrates during germination, raising the protein content of the grain. All these differences depend on the metabolism of the grain; in addition, the permeability of the seed coat to water depends on its composition. For instance, the presence of phenols and flavonoids can lower permeability ( Bewley *et al.*, 2012; Aguilar *et al.*, 2020). The previous behavior is contrary to that reported by Hernández-Becerra *et al.* (2020), with two maize varieties, where the protein content in the meals does not undergo changes throughout malting, which could be interpreted to mean that proteins can be degraded to polypeptides, peptides and amino acids that provide substances for de novo protein synthesis in the growing embryo, but all of them contribute to total nitrogen. Oseguera-Toledo *et al.* (2020) reports an increase in protein content in sorghum malt, indicating that enzymes can contribute to the increase in protein and that this process continues during fermentation, obtaining in both cases raw materials with higher nutritional value. This increase in protein content in pea would be important attribute that affects yeast nutrition, fermentation, foam stability and beer flavor, thinking about this possibility of use ( Alfeo *et al.*, 2018). Table 1 shows the changes on amylose content over the germination time. Germination is a kinematic process governed by enzymatic processes α and β-amylose hydrolyzed during of germination. In this table, the apparent amylose does not significantly change, but when amylopectin degrades, it transforms into amylose, so the values at the end are the same.

### Morphological analysis of isolated starches

**Figure 3.**
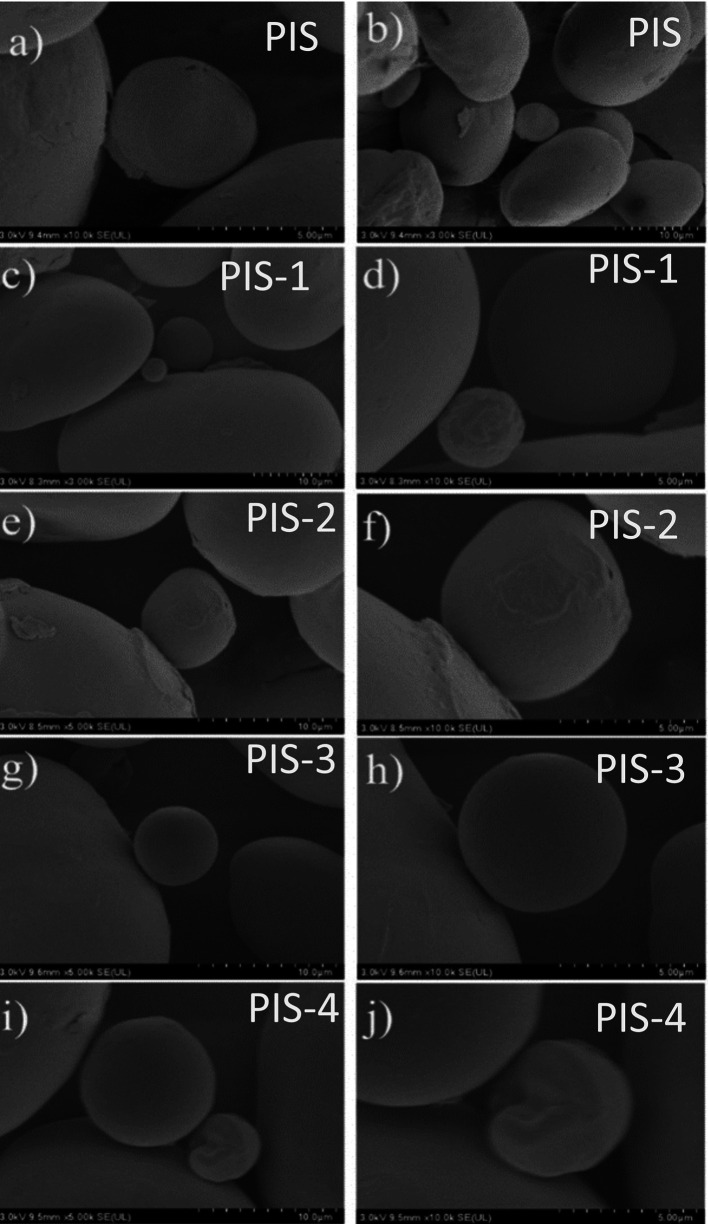
SEM images of the pea isolated starches along the germination, PIS, PIS-1, PIS-2, PIS-3, and PIS-4.

The SEM images of the PIS and isolated starches obtained from day 1 to day 4 of germination (PIS-1 to PIS-4) are shown in Figure 3a to j, where Figure 3a and b represent the PIS, Figure 3c and d represent day1 (PIS-1), Figure 3e and f represent day2 (PIS-2), Figure 3g and h represent day 3 (PIS-3), and Figure 3i and j represent day 4 (PIS-4) of germination. No significant alterations to the starch surface were seen during pea germination. However, there is evidence of some protein and fat removal. As was reported for barley, α and β-amylase form micro-holes during this stage of germination ( Contreras-Jiménez *et al.*, 2018), for corn ( Hernández-Becerra *et al.*, 2020) and ( Oseguera-Toledo *et al.*, 2020).

The starch granules, which coincide with those described by Gao *et al.* (2022), have oval and kidney shapes with a few surface indentations. But with few changes during the germination process. Some starch granules presented only small changes on their surface, observing some indentations or rough surfaces, where an enzymatic attack could have occurred. All of this contrasts with what Gao *et al.* (2022), which states that after the first day of germination, the majority of the starch granules had notches on the surface, with obvious pores and more undulations on the surface of the germinated starch granules as germination progressed, changes that affect the other physical and thermal properties. These changes were produced by the enzymes responsible for amylolysis, in 2 varieties of pea.

### Particle size distribution

The particle size distribution of starch granules D10, D50, and D90 indicate that 10, 50, and 90% of the starch granules have a smaller diameter than this value ( Table 2). In general, D50 was used to describe the mean diameter. The D50 of the starch granules ranged from 53.72±4.65 μm to 56.8±3.41 μm.

**Table 2.** Functional properties and particle size distribution of germinated pea starch.

Sample	WAI (g gel/g sample)	WSI (%)	SP (%)	D10 (μm)	D50 (μm)	D90 (μm)
**PIS**	4.188±0.037 ^c^	2.628±0.244 ^c^	4.234±0.034 ^c^	20.475±1.875 ^a^	53.725±4.657ª	166.925±15.734 ^cd^
**PIS-1**	3.51±0.133 ^b^	1.648±0.139 ^ab^	3.533±0,133 ^b^	20.975±2.278 ^b^	53.475±4.168ª	164.15±15.427 ^c^
**PIS-2**	3.424±0.058 ^b^	1.764±0.12 ^b^	3.448±0.059 ^b^	21.5±3.579 ^bc^	54.475±3.161 ^b^	155.9±8.934ª
**PIS-3**	3.273±0.05 ^a^	1.377±0.111 ^a^	3.29±0.051 ^a^	21.4±3.254 ^bc^	56.2±3.106	157.325±15.765 ^b^
**PIS-4**	4.287±0.085 ^c^	2.629±0.259 ^c^	4.334±0.085 ^c^	23.125±1.706 ^d^	^c^56.8±3.414 ^c^	154.75±22.743ª

The values correspond to average of functional properties and standard deviation.

Different letters in the same column show significant differences using α = 0.05.

In general, statistically significant differences were found in the average diameter in each of the percentiles (p<0.05), presenting a slight increase in the average diameter with advancing germination time, in percentiles D10 and D50, coinciding with what was reported by Gao *et al.* (2022), with starches from two pea varieties. In the D90 percentile, the average diameter of the starch decreased, which could be an effect of the enzymes in the germination process, which could penetrate inside the granules and hydrolyze from the helium region to the outside. This could result in the formation of surface pores, surface erosion, sponge-like erosion and degraded granules, for the grains where there is enzyme activity, which could be occurring superficially in the pea starch granules according to the SEM images obtained in this study, although no enzymatic activity is observed ( Li *et al.*, 2017).

### Structural characterization of isolated starches

**Figure 4.**
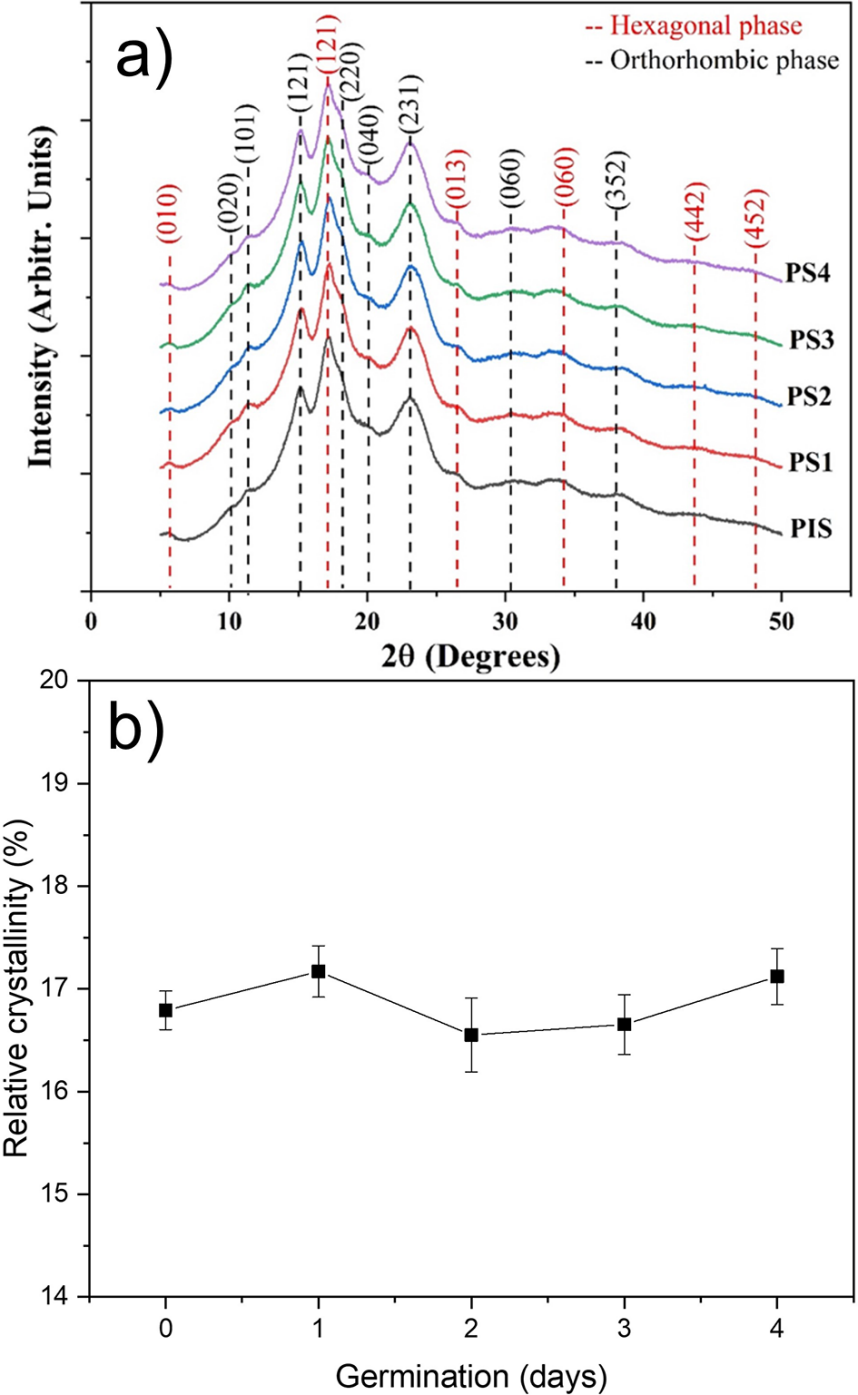
a) X-ray diffraction patterns of pea isolated starch (PIS) and PIS-1, PIS-2, PIS-3, and PIS-4 obtained during germination, b) changes in the apparent crystallinity.

The X-ray diffraction patterns for the PIS and starches obtained from peas subjected to germination for four days are shown in Figure 4. For the indexing of these patterns, tables provided by Rodríguez-García et al. (2021) were used. It was found that pea starches, with and without germination, contain a mixture of hexagonal and orthorhombic glucopyranose crystals, confirming that starches exhibiting this combination are classified as C-type ( Gao et al., 2022). These patterns reveal that these crystalline structures do not undergo any alteration during germination. Rodríguez-García et al. (2021) indexed the orthorhombic structure for A-type starch and the hexagonal structure for B-type starch, and the dashed lines in this figure correspond to the indexation of both structures. Gao et al. (2022) studied the structural transformation during the germination process in two pea varieties. Their results indicated that, during germination, the crystalline structures present in starch do not show significant changes; however, they did not index the patterns to confirm that pea starch can be classified as C-type. Liu et al. (2020) reported that different degrees of crystallinity can indicate differences in the chemical structure and composition of starches. However, from a crystallographic point of view, the term “chemical structure” is not meaningful.

The variations in the relative crystallinity percentage as a function of germination day are shown in Figure 4b. The relative crystallinity is around 17%, and these results show that enzymatic activity during germination does not induce structural alterations. The slight variations in this parameter may be related to the sample’s moisture content. While Rodríguez-García et al. (2021) assert that crystallinity is directly related to orthorhombic and hexagonal crystalline structures, Rupollo et al. (2011) noted that relative crystallinity is typically associated with the content and average chain length of amylopectin, the internal orientation, the degree of interaction of the double helix, and the moisture content of starch granules. Of course, the determination of these patterns was performed at very low moisture (approximately 10%). This suggests that the outward diffusion of bound water into these nanocrystalline formations may alter their crystalline structure ( Kim et al., 2008; 2010; Gong et al., 2016).

### Thermal properties

**Figure 5.**
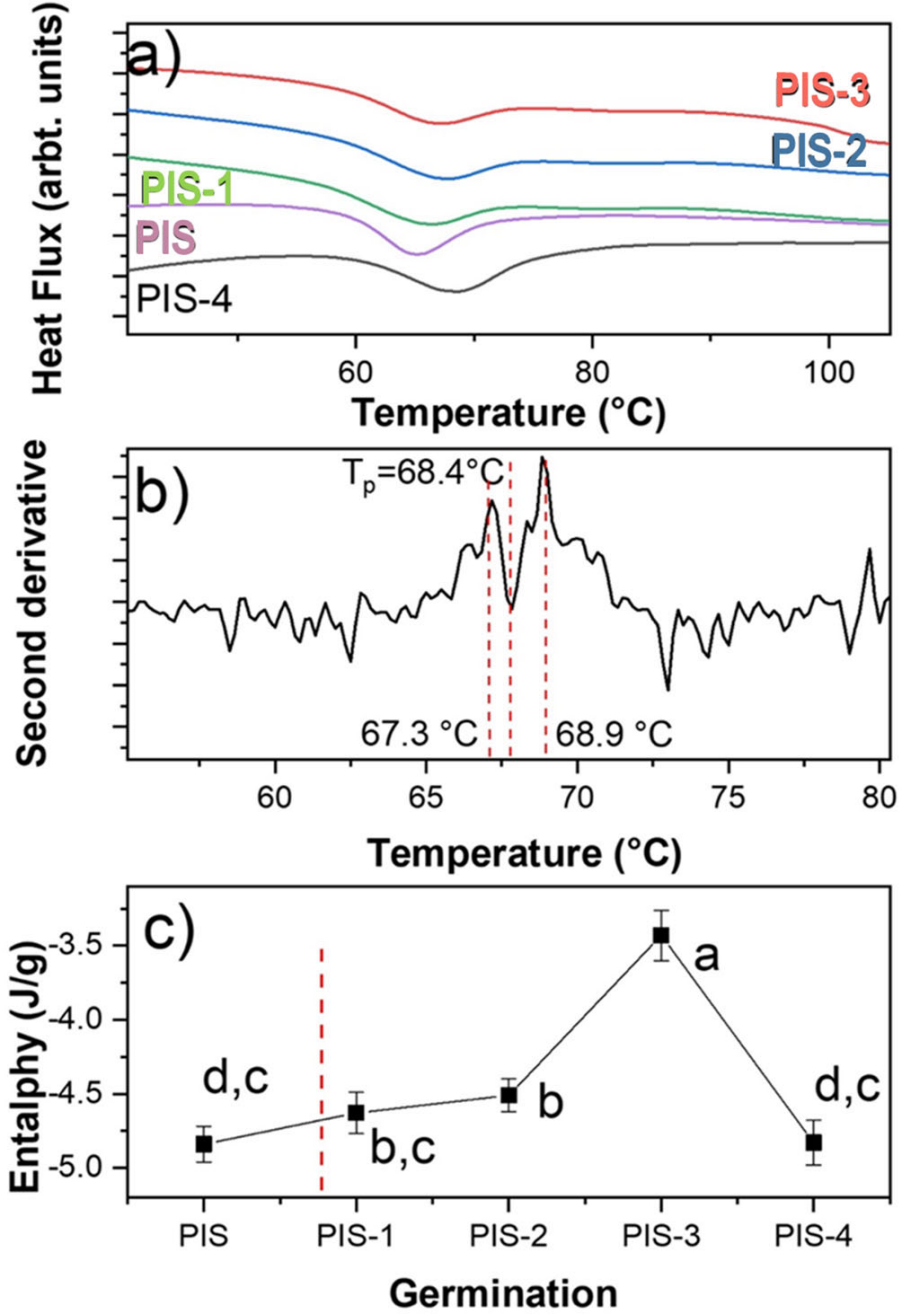
a) DSC thermograms of the PIS, PIS-1, PIS-2, PIS-3, and PIS-4, b) a characteristic second derivative for sample PIS-4, and c) changes in the enthalpy as a function of the germination day. The values correspond to average of enthalpy and standard deviation. Different letters in panel c show significant differences using α = 0.05.

Figure 5a displays the DSC thermograms of native starch (PIS) and starches with different germination times (PIS-1 to PIS-4). This figure shows an endothermic thermal transition for PIS around 65.3°C, which corresponds to an ordered to disordered transition produced by the solvation of the orthorhombic nanocrystals and corresponds to the gelatinization. The gelatinization is a second order thermal event (irreversible transition).

When employing the second derivative criterion presented in Figure 5b, it is evident that gelatinization corresponds to two thermal events even if a preliminary inspection of the endothermic transition does not indicate the presence of more than one transition. These events are directly related to the solvation of orthorhombic and hexagonal nanocrystals, which are what make up this starch according to Figure 4a, which demonstrated this. The first occurrence involves the solvation of the orthorhombic structure, which contains eight water molecules in its structure, and the second involves the solvation of the hexagonal structure, which has 32 water molecules in its crystalline structure ( Esquivel-Fajardo *et al.*, 2022).

For the nanocrystals solvation, a step known as gelatinization must occur. An ordered-disordered transition has been connected to starch gelatinization ( Zobel *et al.*, 1988). There is, however, no mention of the type of structure that is engaged in this transition. The endothermic event can be represented in two steps from a thermodynamic perspective. In the case of pea starch, the orthorhombic and hexagonal nanocrystals absorb energy to raise the temperature, the vibrational states reflect the energy, and the amplitude of the vibrations increases from T _o_ to T _p_. From a quantum perspective, each vibrational state increases its amplitude, and each vibrational mode only reaches its maximum amplitude at T _p_.

The endotherm peak returns to the initial level because each vibrational state is obliterated sequentially from T _p_ to T _e_. The ordered system is represented by the first stage of the endothermic transition, while the disordered system is represented by T _p_. It is evident from a close examination of Figure 4a that neither orthorhombic nor hexagonal nanocrystals alter during germination.

### Vibrational analysis

**Figure 6.**
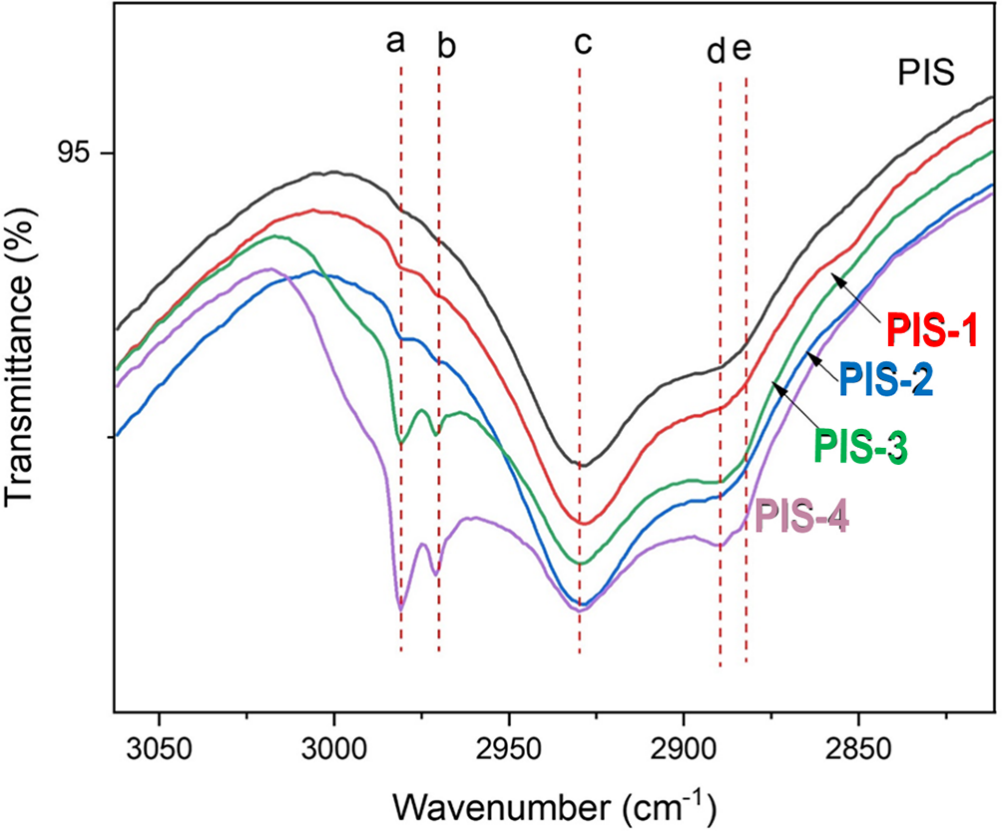
IR spectra for pea isolated starch (PIS) and isolated starches during germination time.

Figure 6 shows the changes in the vibrational spectrum of isolated native starch and germinated starches. In this section, attention was focused on the region in which the vibrational modes were changed due to the germination process. The description of the well-known bands is as follows: a germination split signal from day 1 to day 4 located at 1240, 1407 and 1456 cm ^-1^ of the C-H group stretch corresponds to carbohydrates were observed.

According to the results presented, the most important changes occurred during day 3 and day 4 of germination. At this time, the enzymatic process initiates the modification in the starch granules. No significant changes are shown after soaking. This can be explained because during soaking the seed hydrates and some starch granules can swell. At the same time, an internal enzymatic process in the pea is activated, although the granules remain intact. Germination modified the chemical bonds of the amylose and amylopectin to produce sugar chains that will be used by the grain as a source of energy and carbon for growth. Changes in the signal corresponding to –OH were found at 1275 cm ^-1^, possibly showing the modifications of the glycosidic linkages in α(1–4) and α(1–6). The modification of the signal in the different stages of malting demonstrates the changes in the structure of the starch ( Oseguera-Toledo *et al.*, 2020).

In turn, the presence of characteristic vibrations for functional groups typical of starch could be evidenced (-CH-, -CH _2_-, C-OH, and groups -OH) to wavelengths to 3100, 2915, 2850, 1680, 998, and 855 cm ^-1^ ( Quintero-Castaño *et al.*, 2020). The bands at 1640 and 1545 cm ^−1^ can be attributed to the modification that proteins undergo during germination, which causes the vibration of the released amino acids, especially in the Amide I bands (1650 cm ^−1^), which corresponds to the C=O stretching, and Amide II (1545 cm ^−1^), which corresponds to the bending vibration of the N-H bond and, to a lesser extent, to the stretching of the C-N bond ( Contreras-Jiménez *et al.*, 2018). Other authors have reported similar bands in the germination process of the pea. Xing *et al.* (2021) showed changes in the malting process of quinoa starch, mainly due to 1047 y 1022 cm ^-1^ associated with the fingerprint region of the compound product of the vibration of bonds C-O-H. Chinma *et al.* (2022) reported significant changes to 2930 cm ^-1^ for germinated pea fluor. These changes are associated with changes in the C-H vibrations typical of amylose and amylopectin chains.

The band at 2980 cm ^−1^ (a) with absorber water ( Chang *et al.*, 2014). 2970 cm ^-1^ is due to CH _2_ stretching vibration (b), 2930 cm ^-1^ (c) to υ(C-H ( Oniszczuk *et al.*, 2019); the band at 2890 cm ^-1^(d) to υst(C-H) or υ(C-H) in CH _2_/CH _3_ group, and the band at 2882 cm ^-1^ (e) corresponds to υst(C-H) or υ(C-H) in CH _2_/CH _3_ group. The expression of these IR bands are an indicative of the amylose or amylopectin debranching produce more vibrational centers (quantum states).

### Pasting properties

**Figure 7.**
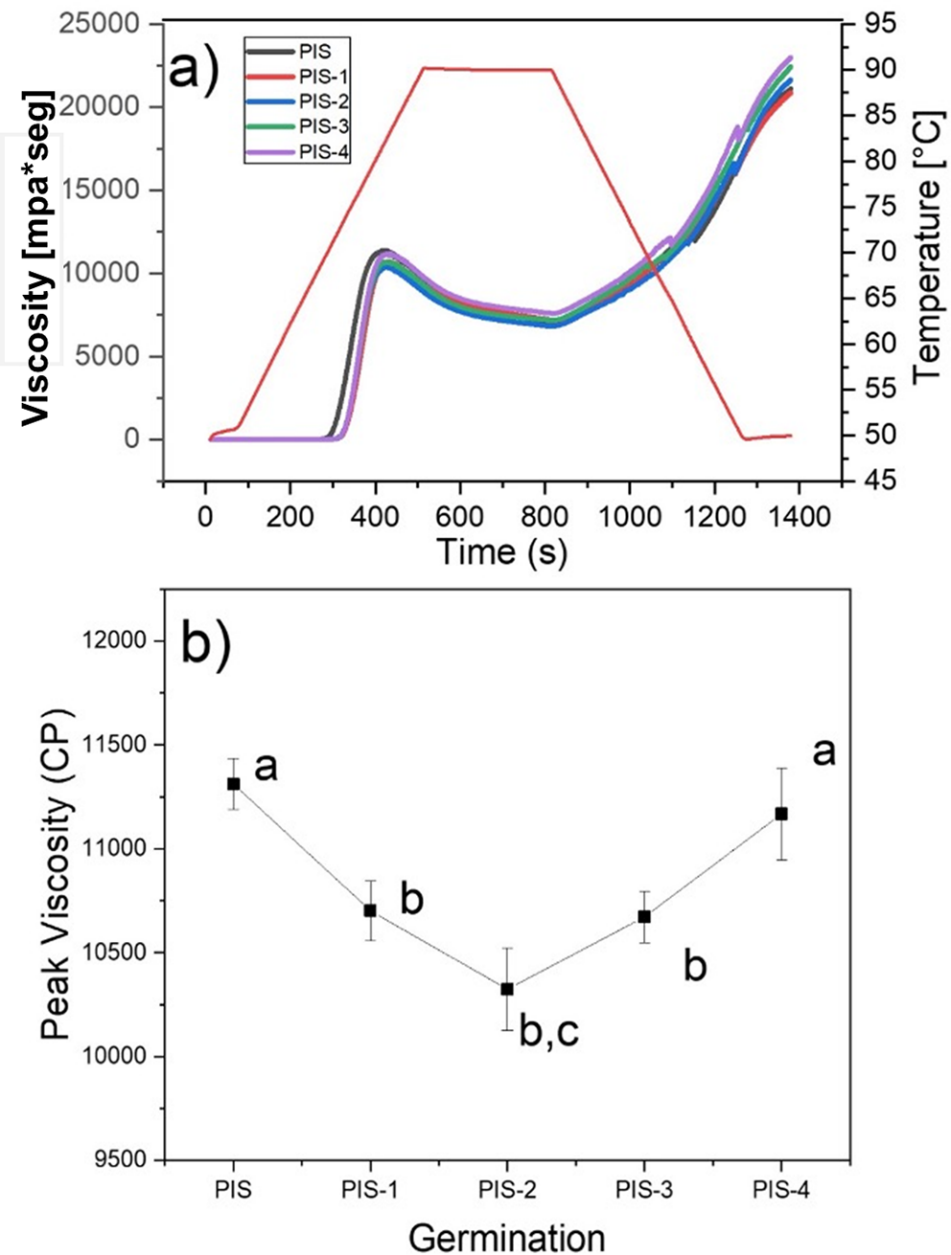
a) Pasting profiles of PIS, PIS-1, PIS-2, PIS-3, and PIS-4 and b) changes in the peak viscosity as a function of the germination. Different letters in panel b show significant differences using α = 0.05.

The viscosity profiles of the PIS and germinated pea starches PIS-1, PIS-2, PIS-3, and PIS-4 are shown in Figure 7a, and the changes in maximum viscosity during germination are shown in Figure 7b. It was found that during germination time there were no significant changes in the viscosity profiles of the isolated starch, which agrees with the morphological analysis of the starches (SEM), where there were no changes on the grains surface. The behavior is in contrast to that observed in Puma and Palomero corn starches by Hernández-Becerra *et al.* (2020), malted barley by Contreras-Jiménez *et al.* (2018), and sorghum malting by Oseguera-Toledo *et al.* (2020), where it was discovered that during the germination and malting processes, peak and final viscosities decrease where starch is degraded. Indicating that the long chains of amylose and amylopectin were fractionated, producing an increase in reducing sugars and a decrease in apparent viscosity, due to the activation of α-amylases that degrade the amorphous component of starch, obtaining mono and disaccharides used as an energy source during malting, and therefore affecting viscosity and by the interaction of water with the starch crystals, which is corroborated by SEM images, where the starch grains are almost completely broken. X-ray analysis showed that the crystalline structures of the starch remains unchanged throughout the malting process ( Contreras-Jiménez *et al.*, 2014; Oseguera-Toledo *et al.*, 2020). Londoño-Restrepo *et al.* (2018) showed that along with the pasting profile, the corn starch granules are disrupted, and at the end of the peak viscosity they showed that there are not any crystalline compounds, because of the X-ray pattern does not exhibit any crystalline phase. Recently, Esquivel-Fajardo *et al.* (2022) showed that at the end of the pasting profile of isolate avocado starch, some fraction of the crystalline components of the starch retains some grade of crystallinity.

They also go against the findings of Gao *et al.* (2022), who found that as germination time increased, the parameters of the viscosity profiles of starches isolated from two native and germinated pea varieties revealed substantial statistical variations. When germination time was extended, the peak viscosity first rose and then fell, reaching its maximum values on days 3 for Xiwan variety 1 and day 1 for Xiwan variety 2, respectively. According to Xing *et al.* (2021), the peak viscosity of mung bean starch decreased when the germination time was extended to 72 h, while the peak viscosity of quinoa starch decreased as the germination time increased ( Liu *et al.*, 2020). The temperature at which the paste of germinated starches begins to gelatinize is likely to have increased when compared to native starch. According to some authors, this characteristic relates to the initial alteration of starch granules, which is the change from starch suspension in water to gel formation ( Rincón-Londoño *et al.*, 2016). Additionally, the thermal characteristics of starch are proportional to the pasting temperature. The same behavior was reported by Hernández-Becerra *et al.* (2020) for Puma and Palomero corn starches, which presented an increase in pasting temperature.

At the same time, native and germinated pea starches discovered in this study displayed higher peak and final viscosities than those obtained in malted Puma and Palomero corn ( Hernández-Becerra *et al.*, 2020); in malted barley ( Contreras-Jiménez *et al.*, 2018); and in two varieties of pea ( Gao *et al.*, 2022). According to the results of starch morphological analysis (SEM) and X-ray, higher viscosities are the result of less enzymatic modification of pea starches. These differences may be related to the amylose-amylopectin relationship, aggregation of starch grains, the fine structure of amylopectin, and the size of starch granules ( Gao *et al.*, 2022). At the same time, once native and germinated pea starch paste was cooled, their final viscosities often increased by a factor of two, from 10.000 mPa*seg to more than 20.000 mPa*seg.

It is important to highlight the possible presence of fat and protein residues in isolated pea starches at different germination times, which could influence the greater resistance of the starch granules to swelling and rupture, due to the formation of lipid-amylose and/or amylopectin complexes that increase the gelatinization onset temperature (T _g_), increasing the integrity of the starch granule, where lipids act as adhesive material and contribute to the formation of granules that fuse to become compound granules ( Xu et al., 2019; Joshi et al., 2013).

Additionally, because of heating, protein residues are denatured and deformed, which encourages protein aggregation and the formation of disulfide bonds between proteins. These effects contribute to protein-protein and protein-starch interactions, which by binding exogenous proteins to the starch grains and preventing water from diffusing through it, affect T _g_. Denatured proteins also reveal several hydrophilic groups (like -COOH, -NH _2_, -OH, and -SH) and hydrophobic amino acids (like Pro, Leu, Phe, Val, and Ile), which may all bind to the surfaces of starch granules by hydrogen bonding or hydrophobic interactions, increasing T _g_. A similar effect was seen by Ribotta *et al.* (2012), who found that pea protein isolate (PPI) dramatically changed how corn and cassava starches pasted during the heating-cooling cycle, resulting in an increase in viscosity. In the samples of maize starch, the pasting temperature was lowered by the addition of PPI. Conversely, the inclusion of PPI enhanced the PV (peak viscosity), FV (final viscosity), BD (breakdown), and SB (setback) of both starch pastes during the heating-cooling process. The pasting temperature (PT) also increased marginally with protein isolates in cassava samples.

### Functional properties of starches

Table 2 shows the results of the functional properties and the type of gel formed by pea isolated starch (PIS) and for day 1 to day 4 (PIS-1 to PIS-4) as a function of the germination (see Figure 7b), where it is initially observed that although in general there are statistically significant differences between WAI, WSI, and SP (p<0.05) and germination time, these are not large enough. At the same time, when comparing the functional properties, with respect to cassava starch, the report reference values are: WAI varies between 0.82 and 15.52 g gel/g sample; WSI: 0.27-12.32% and SP: 0.79 and 15.45% ( Aristizábal and Sánchez, 2007; Wang *et al.*, 2022). It can be concluded that pea starch has medium quality characteristics by developing medium WAI and SP values, and low values in WSI. Contreras-Jiménez *et al.* (2018), with isolated quinoa starch and Ozcan and Jackson (2005), with corn starch, reported values of 2.35±0.03 g gel/g starch and 1.77 g gel/g starch in WAI, respectively, low values with respect to pea starch ( Table 2).

Regarding WSI, pea starch reported less than 2.63%, much lower than the figures reported by Contreras-Jiménez *et al.* (2018) for quinoa starch (4.56±0.0%). This finding indicates that pea starch presents an important number of soluble compounds, which can be included as fiber and other carbohydrates different from starch. In relation to the swelling power (SP) of pea starch, higher values were found with respect to quinoa starch 2.98±0.07% ( Contreras-Jiménez *et al.*, 2018); this last behavior may be related to viscosity, as the higher the SP values, the higher the viscosity value is expected, which is related to the type of gel (solid) obtained with pea starch.

## Conclusions

In the kinematic process of germination, the timing of the distinct starch components is altered. The surface of the starch granules is not altered because of the enzymatic action during germination. While the protein content of the pea grain increased, this could be due to the anabolism of some proteins from reserve carbohydrates and/or enzymatic means. Fat was one of the components that decreased during germination from 2.08±0.06% to 0.53±0.05%, indicating that the fat is involved in several metabolic degradation processes that demand energy and facilitate the emergence of the radicle and hypocotyl in the pea grain.

Amylose and amylopectin are fractionated during the early stages of germination by an enzymatic assault, although orthorhombic and hexagonal nanocrystals are unaltered. More in-depth research is required about the little enzymatic activity of a α and β- amylases on the degradation of starch during pea germination. For longer periods of time, these nano crystals serve as the primary energy source for the germination stage. Pea cannot be used for fermented beverages, as evidenced by the pasting behavior of the flours at rapid germination times.

## CRediT authorship contribution statement

Víctor D. Quintero, Juan Carlos Lucas and Luisa F. Castañeda Formal analysis.: Conceptualization, Writing - review & editing. Mario E. Rodríguez, Juan C. Lucas. Investigation, Writing - review & editing. E. Rodríguez-García Mario: Resources, Writing - review & editing. Victor D. Quintero and Juan Carlos Lucas: Methodology, Resources, Writing - review & editing. Juan Carlos Lucas: Project administration.

## Data Availability

figshare: Data repository of the article titled “Morphological, structural, chemical, vibrational, thermal, pasting, and functional changes in pea starch during germination process”, https://doi.org/10.6084/m9.figshare.23605389.v1 ( Lucas *et al.*, 2023) This project contains the following underlying data:
- RVA PEA: RVA results. - Properties starch PEA: results of functional properties, amylose, proximal. - X-ray patterns: x-ray results. - IR stach: FTIR results native and germinated starches day 1-4. - DCS PEA: DSC results native and germinated starches day 1-4. - SEM Strach: native 0002-0013; Day 1_0001-0010; Day 2_0001-0004; Day 2_0001-0004; Day 4_0001-0009. Data are available under the terms of the Creative Commons Zero ’No rights reserved” data waiver (CC0 1.0 Public domain dedication).
