# Supplementary material for: Morphological, structural, chemical, vibrational, thermal, pasting, and functional changes in pea starch during germination process
Source: F1000Res. 2025 Sep 11;12:940. Originally published 2023 Aug 7. [Version 2] doi: 10.12688/f1000research.136568.2 (PMC12504935; doi:10.12688/f1000research.136568.2)
Supplement: Peer review history

# Reviewer report: Reviewer response for version 2

**DOI:** 10.5256/f1000research.186970.r417776

**Published:** 2025-10-07

**Authors:** Sunday Samuel Sobowale, Joy Agbawodike

**Affiliations:** 1 Mountain Top University, Prayer City, Nigeria; 2 Department of Food Science and Nutrition, University of Minnesota Twin Cities (Ringgold ID: 311837), Saint Paul, Minnesota, USA

- In the abstract section, briefly mention the pea variety used and summarize the major methods (scanning electron microscopy, X-ray diffraction, functional and viscosity tests).

 This will help readers know what was actually measured and how. Include key quantitative data, such as the actual value for gelatinization onset temperature, and the range or mean values for water absorption and swelling power.

 - The authors alternates between *peas*, *beans*, and *grains* Please standardize terminology throughout (e.g., consistently use “peas” or “pea grains”)

 -

Is the work clearly and accurately presented and does it cite the current literature?

Yes

If applicable, is the statistical analysis and its interpretation appropriate?

I cannot comment. A qualified statistician is required.

Are all the source data underlying the results available to ensure full reproducibility?

Yes

Is the study design appropriate and is the work technically sound?

Yes

Are the conclusions drawn adequately supported by the results?

Yes

Are sufficient details of methods and analysis provided to allow replication by others?

Yes

Reviewer Expertise:

Food structure, Food engineering, Structure-function relationships, Functional and physiochemical properties

We confirm that we have read this submission and believe that we have an appropriate level of expertise to confirm that it is of an acceptable scientific standard.

# Reviewer report: Reviewer response for version 2

**DOI:** 10.5256/f1000research.186970.r413283

**Published:** 2025-09-24

**Authors:** Mária Herminia Ferrari Felisberto

**Affiliations:** 1 Food Technology, Federal University of Vicosa, Viçosa, State of Minas Gerais, Brazil

The authors made all the suggested changes; however, there are two points that need to be revised before indexing:

 1 - The sentence: "As reported for barley, α- and β-amylase form microholes during this stage of germination (Contreras-Jiménez et al., 2018), for corn (Hernández-Becerra et al., 2020), and (Oseguera-Toledo et al., 2020)." doesn't make sense where it's located...it needs to be contextualized. Re-evaluate.

 2 - "The description of the well-known bands is as follows: a germination split signal from day 1 to day 4 located at 1240, 1407, and 1456 cm-1 of the C-H group stretch corresponds to carbohydrates were observed. Please re-evaluate, as the lengths mentioned do not even appear in the image used. Please re-evaluate."

Is the work clearly and accurately presented and does it cite the current literature?

Yes

If applicable, is the statistical analysis and its interpretation appropriate?

Yes

Are all the source data underlying the results available to ensure full reproducibility?

Yes

Is the study design appropriate and is the work technically sound?

Yes

Are the conclusions drawn adequately supported by the results?

Yes

Are sufficient details of methods and analysis provided to allow replication by others?

Yes

Reviewer Expertise:

Cereal, bakery products, starch, fiber

I confirm that I have read this submission and believe that I have an appropriate level of expertise to confirm that it is of an acceptable scientific standard, however I have significant reservations, as outlined above.

# Reviewer report: Reviewer response for version 1

**DOI:** 10.5256/f1000research.149711.r228140

**Published:** 2024-03-05

**Authors:** Lesław Juszczak

**Affiliations:** 1 Department of Food Analysis and Evaluation of Food Quality, University of Agriculture in Krakow, Krakow, Poland

Review on manuscript:

 Morphological, structural, chemical, vibrational, thermal, pasting, and functional changes in pea starch during germination process

 by YJuan Carlos Lucas-Aguirre , Víctor Dumar Quintero-Castaño,

 Luisa Fernanda Castañeda-Cano, Mario Enrique Rodríguez-García submitted to F1000Research journal.

 In the manuscript submitted for comments the authors studied the impact of germination time on morphological, structural, thermal and functional properties of starch. Generally, the manuscript is interesting but before acceptance should be corrected and supplemented in some points.

 Detailed recommendation:

 Title, page 3 – what do the authors understand by the vibrational properties of starch?

 page 3, lines 11-12 – literature sources should be added,

 page 3, line 18 – what does "storage proteins" mean?

 page 3, line 38 – properties of what?

 page 4, lines 1 and 15 – origin country should be added,

 page 4, line 20 – model, producer and origin country of spectrophotometer should be added,

 page 5, line 22 and page 11 – better will be: FTIR analysis, how was the sample prepared?

 page 5, line 32 – what parameters were determined?

 page 5, line 35 – Anton Paar is an Austrian company,

 page 5, line 38 – what parameters were determined?

 page 5, line 38 – rpm is not a unit of frequency,

 page 5, line 42 – instead of rpm, the centrifugal force should be provided,

 Table 2 – an explanation of the meaning of superscript letters should be provided in the legend,

 page 9, line 26 and figure 4b –  in the methodology, the authors do not mention the calculation of relative crystallinity,

 Figure 7 – viscosity should be expressed in SI unit,

 page 13 – instead of cP, the SI unit should be used,

 page 14 – is this a legend for table 2 on page 9?

Is the work clearly and accurately presented and does it cite the current literature?

Yes

If applicable, is the statistical analysis and its interpretation appropriate?

Yes

Are all the source data underlying the results available to ensure full reproducibility?

Partly

Is the study design appropriate and is the work technically sound?

Yes

Are the conclusions drawn adequately supported by the results?

Yes

Are sufficient details of methods and analysis provided to allow replication by others?

Partly

Reviewer Expertise:

food analysis, carhohydrtes, starch, honey, food rheology,

I confirm that I have read this submission and believe that I have an appropriate level of expertise to confirm that it is of an acceptable scientific standard.

# Response

**Published:** 2025-09-03

**Authors:** Juan Carlos Lucas-Aguirre

**Affiliations:** Facultad de Ciencias Agroindus, Universidad del Quindío, Armenia, Quindío, Colombia

Title, page 3 – what do the authors understand by the vibrational properties of starch?

**A//:** The vibrational properties by FT-IR of starch refer to the characteristic vibrations of the chemical bonds within the starch molecule that absorb infrared light at different frequencies. These vibrations, such as the stretching and bending of C-H, O-H, and C-O bonds, generate unique absorption patterns that can be used to identify and characterize different types of starch and their modifications.

 Page 3, lines 11-12 – literature sources should be added,

**A//:** It was adjusted.

 Page 3, line 18 – what does "storage proteins" mean?

**A//: **The term storage proteins, in the context of the paragraph as it appears there, refers to: Germination, a complex metabolic process during which the lipids, carbohydrates and storage proteins of the seed are broken down to obtain the energy and amino acids necessary for the development of the plant. That is, as reserve proteins.

 Page 3, line 38 – properties of what?

**A//:** It was adjusted by adding: of pea starch

 Page 4, lines 1 and 15 – origin country should be added,

**A//:** It was adjusted.

 Page 4, line 20 – model, producer and origin country of spectrophotometer should be added,

**A//:** It was adjusted.

 Page 5, line 22 and page 11 – better will be: FTIR analysis,

**A//: **It was adjusted.

 How was the sample prepared?

**A//:** It was adjusted by adding: with a resolution of 4 cm−1 and 16 scans per sample. Each starch sample was analyzed in triplicate on different samples and the average spectra were reported

 Page 5, line 32 – what parameters were determined?

**A//:** It was adjusted by adding: determining the temperature and enthalpia of starch gelatinization.

 Page 5, line 35 – Anton Paar is an Austrian company,

**A//:** It was adjusted.

 Page 5, line 38 – what parameters were determined?

**A//:** It was adjusted: determining the peak viscosity and the final viscosity after cooling the paste.

 Page 5, line 38 – rpm is not a unit of frequency,

**A//:** It was adjusted: 3.22 Hz.

 Page 5, line 42 – instead of rpm, the centrifugal force should be provided,

**A//:** It was adjusted:  at 1450 x g (2000 rpm).

 Table 2 – an explanation of the meaning of superscript letters should be provided in the legend,

**A//:** It was adjusted.

 Page 9, line 26 and figure 4b –  in the methodology, the authors do not mention the calculation of relative crystallinity,

**A//:** was adjusted, was addicted: The crystallinity (%) was calculated by normalising the integrated diffracted intensity over the measured 2θ range to the integrated non-coherent intensity. The non-coherent intensity was obtained by subtracting the sharp diffraction peaks from the total diffraction pattern using the software Diffract/AT from Socobin VI.2 (Contreras-Jiménez *et al.*, 2018).

 Figure 7 – viscosity should be expressed in SI unit,

**A//: **The graphic was adjusted

 Page 13 – instead of cP, the SI unit should be used,

**A//: **It was adjusted to mPa-s

 Page 14 – is this a legend for table 2 on page 9?

**A//:** It was adjusted.

# Reviewer report: Reviewer response for version 1

**DOI:** 10.5256/f1000research.149711.r213713

**Published:** 2023-11-01

**Authors:** Mária Herminia Ferrari Felisberto

**Affiliations:** 1 Food Technology, Federal University of Vicosa, Viçosa, State of Minas Gerais, Brazil

The work "Morphological, structural, chemical, vibrational, thermal, pasting, and functional changes in pea starch during germination process" brings innovative and preliminary research into the behavior of starch, extracted from a non-conventional source, over different germination times. However, the work presents some points that need to be improved, or better described/explained, as highlighted below:

- At the conclusion of the work summary, the authors mention lentil starch: I believe this was a typo, as it doesn't make sense in the context.
- In the introduction to the work, when justifying the use of peas, the authors start by addressing soy and then enter the gluten-free category, which doesn't make much sense, since soy does not contain gluten. I suggest that the authors explore this argument further, following the line of unconventional or under-explored sources.
- Still in the introduction, the authors use the term "humidity", which I believe should be replaced by "moisture content".
- In the material and methods, I suggest that the authors maintain the analyzes of germinated grains, following the germination process, maintaining a logical sequence of the steps carried out (go up to the Percentage of grain germination methodology).
- Discussion of the results, in the topic "morphological analysis of isolated stars"... I believe there was an error in the elaboration of the last sentence, as the way it was used it doesn't make much sense.
- Grammatical error in line 8 of the topic "structural characterization of isolated stars". And the last sentence of this item was confusing with all the previous discussion... The evidence indicates or does not indicate type-C. What was the authors' conclusion? I suggest adding some reference that corroborates the conclusion obtained and presented in this sentence.
- There is a grammatical error in the last sentence of the following paragraph.
- There is a grammatical error (typing) in the first sentence of the topic "thermal properties".
- Figures 5 and 6: I suggest that the authors use color legends in the graphs, as the indication of the samples in them is confusing the reader.
- Topic "vibrational analysis" mentions some lengths that are not shown in the Figure. I suggest reevaluating the figure, or explaining the reason.
- "amide vibrations" Could this really be the expression? (second paragraph on page 12).
- Page 13, 4th paragraph: "it is important...." Would this really be a plausible explanation, given the very low protein and fat content that could be present in starch? I suggest adding references to this statement.
- Conclusion topic mentions something about "in-depth research...." what do the authors mean? Longer germination time? Other analyses? Additional analyses?

Is the work clearly and accurately presented and does it cite the current literature?

Yes

If applicable, is the statistical analysis and its interpretation appropriate?

Yes

Are all the source data underlying the results available to ensure full reproducibility?

Yes

Is the study design appropriate and is the work technically sound?

Yes

Are the conclusions drawn adequately supported by the results?

Yes

Are sufficient details of methods and analysis provided to allow replication by others?

Yes

Reviewer Expertise:

Cereal, bakery products, starch, fiber

I confirm that I have read this submission and believe that I have an appropriate level of expertise to confirm that it is of an acceptable scientific standard, however I have significant reservations, as outlined above.

# Response

**Published:** 2025-09-03

**Authors:** Juan Carlos Lucas-Aguirre

**Affiliations:** Facultad de Ciencias Agroindus, Universidad del Quindío, Armenia, Quindío, Colombia

At the conclusion of the work summary, the authors mention lentil starch: I believe this was a typo, as it doesn't make sense in the context.

**A//:** It was adjusted

 In the introduction to the work, when justifying the use of peas, the authors start by addressing soy and then enter the gluten-free category, which doesn't make much sense, since soy does not contain gluten. I suggest that the authors explore this argument further, following the line of unconventional or under-explored sources.

**A//:** It was adjusted. A sentence was added that connects the 2 paragraphs.

 Still in the introduction, the authors use the term "humidity", which I believe should be replaced by "moisture content".

**A//:** It was adjusted

 In the material and methods, I suggest that the authors maintain the analyzes of germinated grains, following the germination process, maintaining a logical sequence of the steps carried out (go up to the Percentage of grain germination methodology).

**A//: **It was adjusted

 Discussion of the results, in the topic "morphological analysis of isolated stars"... I believe there was an error in the elaboration of the last sentence, as the way it was used it doesn't make much sense.

**A//:** With all due respect, we note that the term "isolated stars" does not appear anywhere in the sections mentioned by the Evaluator, nor anywhere in the text of the document. Perhaps we believe this is an error when converting the document to PDF.

 Grammatical error in line 8 of the topic "structural characterization of isolated stars". And the last sentence of this item was confusing with all the previous discussion...

**A//:** With all due respect, we note that the term "isolated stars" does not appear anywhere in the sections mentioned by the Evaluator, nor anywhere in the text of the document. Perhaps we believe this is an error when converting the document to PDF.

 The evidence indicates or does not indicate type-C. What was the authors' conclusion? I suggest adding some reference that corroborates the conclusion obtained and presented in this sentence.

**A//: ** It was adjusted, and the bibliographic reference was added.

 There is a grammatical error in the last sentence of the following paragraph.

**A//:**  It was adjusted.

 There is a grammatical error (typing) in the first sentence of the topic "thermal properties".

  It was fixed; the word “of” was repeated in the first line.

 Figures 5 and 6: I suggest that the authors use color legends in the graphs, as the indication of the samples in them is confusing the reader.

**A//:** The graphics were adjusted.

 Topic "vibrational analysis" mentions some lengths that are not shown in the Figure. I suggest reevaluating the figure, or explaining the reason.

**A//:** Respectfully, as we stated at the beginning of the discussion in this section, the analysis focuses on the vibrational modes where the most changes occurred in the starch during the germination process, but at the same time the results also contain traces of other components such as fats, proteins and fibers, which we named as the evaluator stated, but their presence agrees with the results and analysis of the other tests performed.

 "amide vibrations" Could this really be the expression? (second paragraph on page 12).

**A//:** It was adjusted, looking like this: The bands at 1640 and 1545 cm⁻¹ can be attributed to the modification that proteins undergo during germination, which causes the vibration of the released amino acids, especially in the Amide I bands (1650 cm⁻¹), which corresponds to the C=O stretching, and Amide II (1545 cm⁻¹), which corresponds to the bending vibration of the N-H bond and, to a lesser extent, to the stretching of the C-N bond.

 Page 13, 4th paragraph: "it is important...." Would this really be a plausible explanation, given the very low protein and fat content that could be present in starch? I suggest adding references to this statement.

**A//:** It was adjusted, the following bibliographic citations were added: (Xu et al., 2019; Joshi et al., 2013).

 Conclusion topic mentions something about "in-depth research...." what do the authors mean? Longer germination time? Other analyses? Additional analyses?

**A//: **It was adjusted by adding: More in-depth research is required about the little enzymatic activity of a α and β- amylases on the degradation of starch during pea germination.
